# Icariin displays anticancer activity against human esophageal cancer cells via regulating endoplasmic reticulum stress-mediated apoptotic signaling

**DOI:** 10.1038/srep21145

**Published:** 2016-02-19

**Authors:** Chongxi Fan, Yang Yang, Yong Liu, Shuai Jiang, Shouyin Di, Wei Hu, Zhiqiang Ma, Tian Li, Yifang Zhu, Zhenlong Xin, Guiling Wu, Jing Han, Xiaofei Li, Xiaolong Yan

**Affiliations:** 1 Department of Thoracic Surgery, Tangdu Hospital, The Fourth Military Medical University, 1 Xinsi Road, Xi’an 710038, China; 2 Department of Biomedical Engineering, The Fourth Military Medical University, 169 Changle West Road, Xi’an 710032, China; 3 Department of Thoracic Surgery, Guangdong Provincial Corps Hospital of Chinese People’s Armed Police Forces, Guangzhou Medical University, 268 Yanling Road, Guangzhou 510507, China; 4 Department of Aerospace Medicine, The Fourth Military Medical University, 169 Changle West Road, Xi’an 710032, China; 5 Department of Ophthalmology, Tangdu Hospital, The Fourth Military Medical University, 1 Xinsi Road, Xi’an 710038, China

## Abstract

In this study, we investigated the antitumor activity of icariin (ICA) in human esophageal squamous cell carcinoma (ESCC) *in vitro* and *in vivo* and explored the role of endoplasmic reticulum stress (ERS) signaling in this activity. ICA treatment resulted in a dose- and time-dependent decrease in the viability of human EC109 and TE1 ESCCs. Additionally, ICA exhibited strong antitumor activity, as evidenced by reductions in cell migration, adhesion, and intracellular glutathione (GSH) levels and by increases in the EC109 and TE1 cell apoptotic index, Caspase 9 activity, reactive oxygen species (ROS) level, and nicotinamide adenine dinucleotide phosphate (NADPH) oxidase activity. Furthermore, ICA treatments upregulated the levels of ERS-related molecules (p-PERK, GRP78, ATF4, p-eIF2α, and CHOP) and a pro-apoptotic protein (PUMA) and simultaneously downregulated an anti-apoptotic protein (Bcl2) in the two ESCC cell lines. The downregulation of ERS signaling using eIF2α siRNA desensitized EC109 and TE1 cells to ICA treatment, and the upregulation of ERS signaling using thapsigargin sensitized EC109 and TE1 cells to ICA treatment. In summary, ERS activation may represent a mechanism of action for the anticancer activity of ICA in ESCCs, and the activation of ERS signaling may represent a novel therapeutic intervention for human esophageal cancer.

Esophageal cancer is the sixth leading cause of cancer-related mortality in males and the eighth most common cancer worldwide in females^1^. Based on conservative estimates, approximately 70% of global oesophageal cancer cases occur in China^2^. Esophageal cancer comprises two histological types: esophageal squamous cell carcinoma (ESCC) and esophageal adenocarcinoma (EAD). ESCC, characterized by its remarkable geographic distribution and high-risk areas, especially in China, Japan, India, and Africa, typically originates from squamous cells in the middle or upper third of the esophagus^3^. In contrast, EAD is the primary type of esophageal cancer in Western countries and originates from glandular cells in the lower third of the esophagus and/or at the junction between the esophagus and the stomach^4^. Although treatment and perioperative management have evolved in recent years, including dramatic advances in diagnostics, operative methods, and combination chemo-radiotherapy, the prognosis of patients with esophageal cancer is not satisfactory. The 5-year overall survival rate ranges from 20% to 30% after surgery^5^. Thus, understanding the detailed molecular mechanisms involved in esophageal cancer progression is crucial for the development of novel therapeutic strategies. Less harmful plant-derived natural products occupy a very important position in the field of cancer chemotherapy. Flavonoids are plant polyphenols found in vegetables, fruit, and beverages of plant origin and are well known for their anti-inflammatory, analgesic, and physiologically antipyretic activities^6^. Recently, the antitumor activity of flavonoid glycosides has attracted great attention^7^,^8^.

Icariin (ICA, C33H40O15, molecular weight 676.65 g/mol) is a prenylated flavonol glycoside derived from the medical plant *Herba Epimedii* that exhibits a variety of pharmacological activities^9^,^10^,^11^. It has been previously demonstrated that ICA displays potent antitumor activities in various types of cancer, including breast cancer, human Burkitt lymphoma and liver cancer^12^,^13^,^14^. Recently, Zhang *et al.* reported that ICA protected rat H9c2 cardiac cells from apoptosis by inhibiting endoplasmic reticulum (ER) stress (ERS)^15^, indicating that ICA might exhibit anticancer activity by regulating ERS. However, in the current literature, the effects of ICA on human ESCC and its mechanism of action have not been elucidated.

The ER is a eukaryotic organelle that is essential for the regulation of calcium storage and release and serves as the entrance to the secretory pathway, through which approximately one-third of all cellular proteins traffic en route to their proper intracellular or extracellular location^16^. Numerous environmental, physiological and pathological insults, as well as nutritional imbalance, disrupt the protein folding environment in the ER and cause protein misfolding and accumulation, thereby activating the unfolded protein response (UPR), also referred to as ERS^17^. The outcome of UPR activation involves the transient attenuation of protein synthesis, an increased capacity for protein trafficking through the ER and increased protein folding, transport, and degradation via processes such as ER-associated degradation (ERAD) and autophagy^18^. In mammals, three ER membrane-associated proteins act as ERS sensors: (1) inositol-requiring transmembrane kinase/endoribonuclease 1 (IRE1), (2) the double-stranded RNA (PKR)-activated protein kinase-like eukaryotic translation initiation factor 2α (eIF2α) kinase (PERK), and (3) activating transcription factor 6 (ATF6)^19^. Under normal circumstances, these sensors are maintained in an inactive state due to their binding to the chaperone glucose-regulated protein 78 (GRP78), which forms a large multiprotein complex with a set of other ER molecular chaperones, including the heat shock protein of 90 kDa (Hsp90) ER homolog, Grp94; protein disulfide isomerase; calcium binding protein; and cyclophilin B^20^. During ER stress, increased levels of unfolded substrates lead to the sequestration of GRP78, which frees the sensors to initiate UPR signaling^21^. For example, PERK ameliorates ERS through phosphorylation of the translation initiation factor eIF2a. This induces a generalized decrease in protein synthesis while also promoting the translation of a subset of UPR target proteins, including the transcription factor ATF4, which induces expression of the transcription of C/EBP homologous protein (CHOP), eventually enabling the recovery of protein translation^22^. In parallel, CHOP promotes oxidative protein folding in the ER, but this increased formation of disulfide bonds can contribute to worsening cellular stress through the generation of reactive oxygen species (ROS)^23^. The successful neutralization of the activating stress results in cell survival, whereas the failure to neutralize this stress induces cell death. Moreover, eIF2α generally curtails translation in response to a wide array of cellular stresses while activating stress-induced gene expression programs. eIF2α performs important and often essential functions in response to infection, proteotoxicity and cancer^24^. The phosphorylation of eIF2α protects cells by reducing the general rate of protein synthesis and biases the translation initiation machinery toward the translation of mRNAs encoding genes that play roles in stress responses^25^. CHOP also participates in the initiation of apoptosis and organ regeneration^26^. In many ERS pathways, the PERK, IRE1, and ATF6 signaling axes induce the activation of CHOP, eIF2α, and p53 upregulated modulator of apoptosis (PUMA, a member of the Bcl2 protein family), respectively, to sensitize cells to ERS-induced cell death^27^. The Bcl2 family plays a crucial role in the apoptotic process in various cancer types^28^. It has been suggested that Bcl2 (anti-apoptotic) and PUMA (pro-apoptotic) act as opposing members of the Bcl2 family and that these two molecules ultimately regulate cell death in response to ERS^29^. Previous studies have shown that ICA induces the apoptosis of mouse MLTC-10 Leydig tumor cells by regulating the Bcl2 signaling pathway^30^. Furthermore, adenosine inhibits cell proliferation, increases GRP78 and NF-κB p65 expression and induces apoptosis by CHOP and caspase-4 pathways in EC109 cells, which is involved in the ERS pathway^31^. Importantly, Zhang and colleagues showed that ICA protects rat H9c2 cells from apoptosis by inhibiting ERS^15^. However, the role of ERS signaling in the anti-ESCC activity of ICA has not been examined. In the present study, we assessed the anticancer activity of ICA in ESCC and explored the role of ERS signaling in this activity.

## Results

### Effects of ICA treatment on the viability and apoptosis of human ESCCs and primary esophageal epithelial cell

**Figure 1:**
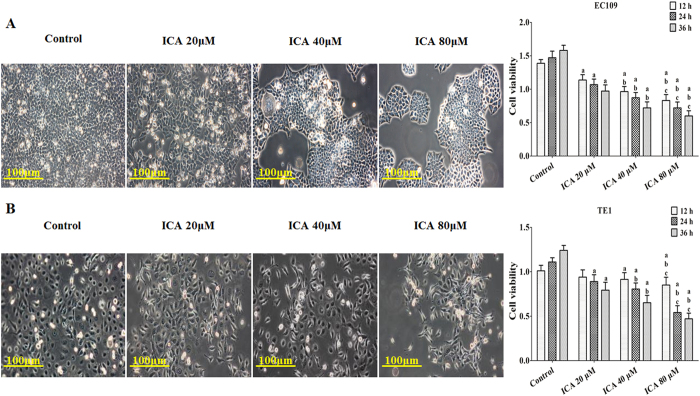
Effect of ICA treatment on the viability and morphology of human ESCC cells. (**A**) EC109 cells were treated with increasing concentrations of ICA (20, 40, or 80 μM) and assessed at different time points (12, 24, and 36 h). The cell viability is expressed as OD values. (**B**) TE1 cells were treated with increasing concentrations of ICA (20, 40, or 80 μM) and assessed at different time points (12, 24, and 36 h). The cell viability is expressed as OD values. The morphology of both of the ESCC lines was observed under an inverted phase-contrast microscope after the cells were treated for 24 h, and images were obtained. Significant cell shrinkage and a decreased cellular attachment rate were observed in the ICA treatment groups. All of the results are expressed as the mean ± SD; n = 6. ^**a**^P < 0.05 *vs.* the control group; ^**b**^P < 0.05 *vs.* the 20 μM ICA-treated group; ^**c**^P < 0.05 *vs.* the 40 μM ICA-treated group.

To investigate whether ICA displays antitumor activity in EC109, TE1, and HET-1A cells, the CCK-8 assay was used to evaluate the effects of ICA on different cells, and the data are shown in Fig. 1. Treatment of EC109 and TE1 cells for 12, 24, or 36 h with 20, 40, or 80 μM ICA inhibited cell viability in a dose- and time-dependent manner. The 50% inhibitory concentrations (IC50s) of ICA at 12, 24, and 36 h were approximately 106.13 μM, 73.65 μM, and 38.59 μM in EC109 cells and 115.29 μM, 76.77 μM, and 42.21 μM in TE1 cells, respectively. Microscopic images (EC109 in Fig. 1A and TE1 in Fig. 1B) indicated that compared with the control treatment ICA treatment resulted in significant cell shrinkage (based on the bar and cellular gap) and reduced the rate of cellular attachment (based on the cell numbers). However, ICA at the same concentrations did not affect the survival of human primary esophageal epithelial HET-1A cell (Supplementary Fig. 2). The results suggest that icariin has promising anti-ESCC activity with low cytotoxic effect on normal esophageal epithelial cell.

**Figure 2:**
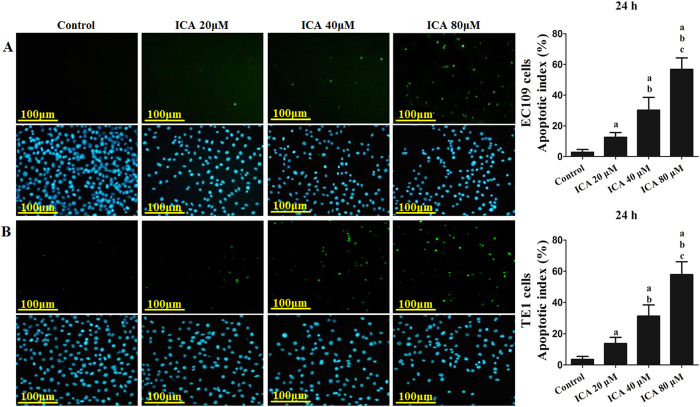
Effect of ICA treatment on the apoptosis of human ESCC cells. After treatment, a dose-dependent increase was observed in apoptosis. (**A**) The apoptotic index of ICA-treated EC109 cells. (**B**) The apoptotic index of ICA-treated TE1 cells. The upper panel showed the apoptotic cells and the lower panel showed the cell nucleus, respectively. All of the results are expressed as the mean ± SD; n = 6. ^**a**^P < 0.05 *vs.* the control group; ^**b**^P < 0.05 *vs.* the 20 μM ICA-treated group; ^**c**^P < 0.05 *vs.* the 40 μM ICA-treated group.

The apoptotic index of ICA-treated ESCC cells was then measured. After treatment with 20, 40, and 80 μM ICA for 24 h, the apoptotic index increased to 12.58 ± 6.07%, 30.21 ± 8.29%, and 56.84 ± 7.39% in EC109 cells and 13.72 ± 3.95%, 31.26 ± 7.22%, and 57.89 ± 8.21% in TE1 cells, respectively (P < 0.05 compared with the control treatment), indicating a dose-dependent increase in the induction of apoptosis (EC109 in Fig. 2A and TE1 in Fig. 2B).

### Effects of ICA treatment on the adhesion and migration of human ESCCs

**Figure 3:**
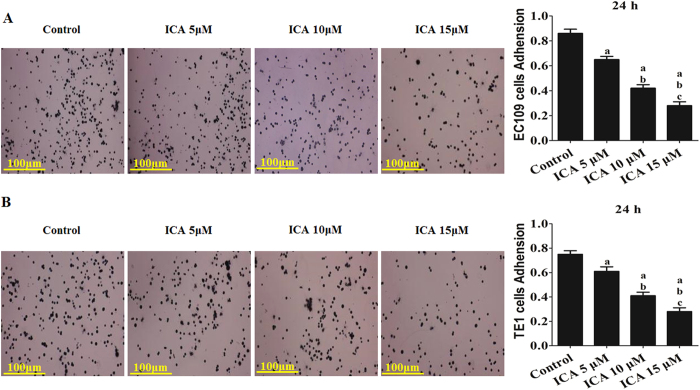
Effect of ICA treatment on the adhesion of human ESCC cells (24 h). (**A**) The adhesion ability of EC109 cells is expressed as an adhesion ratio. (**B**) The adhesion ability of TE1 cells is expressed as an adhesion ratio. The number of adherent cells in the control group was set as 100%. The results are expressed as the mean ± SD; n = 6. ^**a**^P < 0.05 *vs.* the control group; ^**b**^P < 0.05 *vs.* the 5 μM ICA-treated group; ^**c**^P < 0.05 *vs.* the 10 μM ICA-treated group.

**Figure 4:**
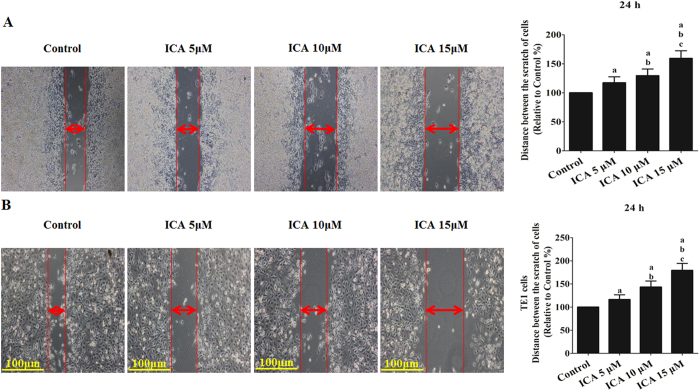
Effect of ICA treatment on the migration of human ESCC cells (24 h). (**A**) The migratory ability of EC109 cells is expressed as the mean distance between the two sides of the scratch. (**B**) The migratory ability of TE1 cells is expressed as the mean distance between the two sides of the scratch. The mean distance in the control group was set as 100%. The results are expressed as the mean ± SD; n = 6. ^**a**^P < 0.05 *vs.* the control group; ^**b**^P < 0.05 *vs.* the 5 μM ICA-treated group; ^**c**^P < 0.05 *vs.* the 10 μM ICA-treated group.

The adhesion and migration of ICA-treated EC109 and TE1 cells were further evaluated. After incubation in ICA (5, 10, or 15 μM) for 24 h, the cell adhesion ratio decreased significantly to 73.71 ± 8.24%, 58.24 ± 6.47%, and 47.23 ± 8.97% of the adhesion of the control in EC109 cells and 76.71 ± 9.57%, 55.29 ± 8.24%, and 45.98 ± 6.72% of the adhesion of the control in TE1 cells, respectively (P < 0.05 compared with the control treatment, EC109 in Fig. 3A and TE1 in Fig. 3B). Similarly, the scratch wound distance significantly increased by 117.26 ± 10.30%, 129.45 ± 11.62%, and 159.23 ± 13.27% in EC109 cells and 116.31 ± 10.36%, 143.41 ± 12.94%, and 179.26 ± 15.14% in TE1 cells, respectively (P < 0.05 compared with the control treatment, EC109 in Fig. 4A and TE1 in Fig. 4B).

### Effects of ICA treatment on Caspase 9 activity, ROS generation, NADPH oxidase activity assay, and GSH levels in human ESCCs

**Figure 5:**
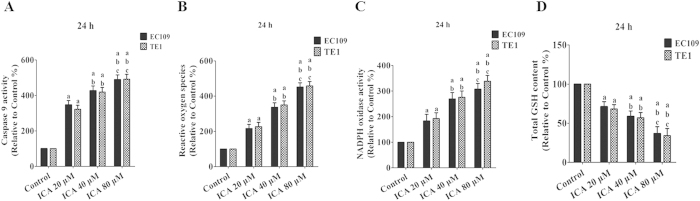
Effect of ICA treatment on Caspase 9 activity, ROS generation, NADPH oxidase activity, and GSH levels in human ESCC cells (24 h). (**A**) The intracellular Caspase 9 activity levels are shown. (**B**) ROS concentrations are shown. (**C**) NADPH oxidase activity is shown. (**D**) Intracellular GSH levels are shown. The three indexes in the control group were defined as 100%. The results are expressed as the mean ± SD; n = 6. ^a^P < 0.05 *vs.* the control group; ^b^P < 0.05 *vs.* the 20 μM ICA-treated group; ^c^P < 0.05 *vs.* the 40 μM ICA-treated group.

After ICA treatment (20, 40, or 80 μM), Caspase 9 activity (Fig. 5A) was significantly increased by 346.84 ± 24.58%, 426.52 ± 27.21%, and 489.23 ± 26.39% in EC109 cells and 321.98 ± 22.56%, 419.21 ± 25.67%, and 492.63 ± 27.66% in TE1 cells, respectively (P < 0.05 compared with the control treatment). Next, intracellular ROS production was analyzed based on the ROS-mediated conversion of the non-fluorescent species 2’,7’-DCFH-DA to the fluorescent species DCFH, which exhibits enhanced fluorescence intensity following the generation of intracellular reactive metabolites. Treatment with ICA (20, 40, or 80 μM) for 24 h induced a dose-dependent increase in ROS generation by 216.46 ± 23.57%, 336.80 ± 25.11%, and 451.80 ± 23.63% in EC109 cells and 224.46 ± 24.67%, 349.80 ± 23.15%, and 457.81 ± 25.18% in TE1 cells, respectively (P < 0.05 compared with the control treatment) (Fig. 5B). The NADPH oxidase system is now widely recognized as a key player in intracellular ROS homeostasis and as one of the major producers of ROS within the cell^32^. After administrated with ICA as above doses, NADPH oxidase activity was increased at a dose-dependent manner from 183.46 ± 25.68%, 269.37 ± 22.58% to 307.67 ± 22.15% in EC109 cells and 192.52 ± 22.86%, 25.82 ± 22.28% to 338.17 ± 3.12% in TE1 cells, respectively (P < 0.05 compared with the control treatment) (Fig. 5C). GSH is the major non-protein thiol in cells and is essential for maintaining cellular redox homeostasis. After treatment with ICA (20, 40, or 80 μM) for 24 h, a dose-dependent decrease in the intracellular GSH levels (to 71.29 ± 6.37%, 58.74 ± 6.98%, and 36.78 ± 8.91% of the levels in controls in EC109 cells and 68.29 ± 5.51%, 56.97 ± 6.84%, and 34.28 ± 8.93% of the levels in controls in TE1 cells, respectively) was observed (P < 0.05 compared with the control treatment, Fig. 5D).

### Effects of ICA on ERS signaling and the Bcl2 protein family in human ESCCs

**Figure 6:**
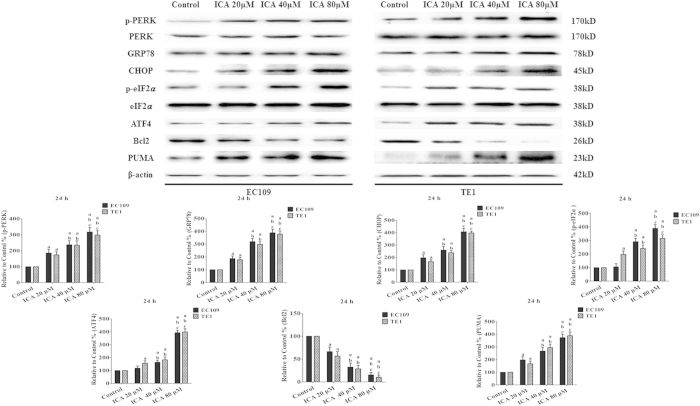
Effect of ICA on ERS signaling and the Bcl2 family of proteins in human ESCC cells (24 h). Representative Western blot results of p-PERK, GRP78, CHOP, p-eIF2α, ATF4, Bcl2, and PUMA are shown. Membranes were re-probed for β-actin expression to show that similar amounts of protein were loaded in each lane. The results are expressed as the mean ± SD; n = 6. ^**a**^P < 0.05 *vs.* the control group; ^**b**^P < 0.05 *vs.* the 20 μM ICA-treated group; ^**c**^P < 0.05 *vs.* the 40 μM ICA-treated group.

To investigate the role of ERS signaling in the anticancer activity of ICA, ERS-related molecules were detected via Western blot in the two ESCC cell lines. Western blots revealed that ICA treatment upregulated GRP78, ATF4, and CHOP expression and p-PERK and p-eIF2α levels in EC109 and TE1 cells in a dose-dependent manner (P < 0.05 compared with the control treatment, Fig. 6). Additionally, we determined whether ICA affects the apoptotic signaling pathway in ESCC cells by measuring the protein expression of Bcl2 protein family members. ICA treatment decreased the expression of Bcl2 and increased the expression of PUMA (P < 0.05 compared with the control treatment, Fig. 6).

### Effects of ICA combined with eIF2α siRNA on cell viability, Caspase 9 activity, ROS generation, NADPH oxidase activity, and p-eIF2α levels in human ESCCs

**Figure 7:**
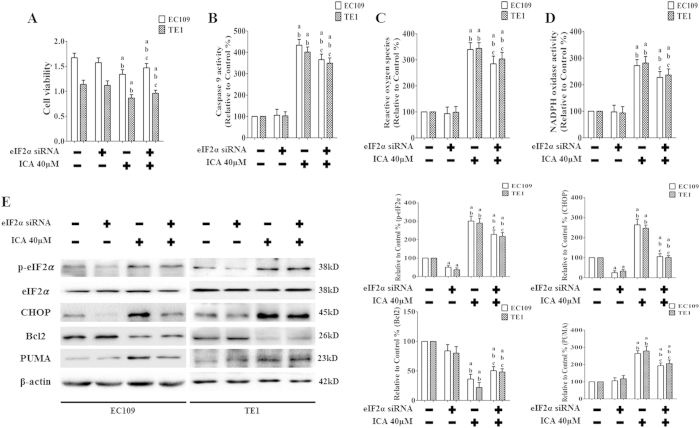
Effect of ICA combined with eIF2α siRNA on cell viability, Caspase 9 activity, ROS induction, NADPH oxidase activity, and p-eIF2α levels in human ESCC cells. (**A**) Viability is shown as OD values. (**B**) The intracellular Caspase 9 activity levels are shown. (**C**) ROS concentrations are shown. (**D**) NADPH oxidase activity is shown. The two indexes in the control group were defined as 100%. (**E**) Representative Western blot results of p-eIF2α, CHOP, Bcl2, and PUMA are shown. Membranes were re-probed for β-actin expression to show that similar amounts of protein were loaded in each lane. The results are expressed as the mean ± SD; n = 6. ^a^P < 0.05 *vs.* the control siRNA group; ^b^P < 0.05 *vs.* the eIF2α siRNA-treated group; ^c^P < 0.05,*vs.* the control siRNA + ICA 40 μM-treated group.

eIF2α siRNA was used to explore the effect of downregulating ERS signaling on the antitumor activity of ICA *in vitro*. ESCC cells were first transfected with eIF2α siRNA and then treated with ICA (40 μM) for an additional 24 h. Transfection with eIF2α siRNA significantly decreased p-eIF2α levels in EC109 and TE1 cells (P < 0.05 compared to transfection with the control siRNA, Fig. 7E). The combination of eIF2α siRNA and ICA significantly increased cell viability (Fig. 7A), decreased Caspase 9 activity (Fig. 7B) and reduced the generation of ROS and NADPH (Fig. 7C,D) (P < 0.05 compared with the combination of control siRNA and ICA); however, eIF2α siRNA alone did not affect cell viability, Caspase 9 activity, ROS generation, or NADPH oxidase activity compared with the control siRNA (P > 0.05). In addition, Bcl2 was further upregulated by co-treatment with ICA and eIF2α siRNA, whereas PUMA was further downregulated by co-treatment with eIF2α siRNA and ICA (P < 0.05 compared with the control siRNA and ICA co-treatment, Fig. 7E).

### Effects of ICA combined with THA on cell viability, Caspase 9 activity, ROS generation, NADPH oxidase activity, and p-eIF2α levels in human ESCCs

**Figure 8:**
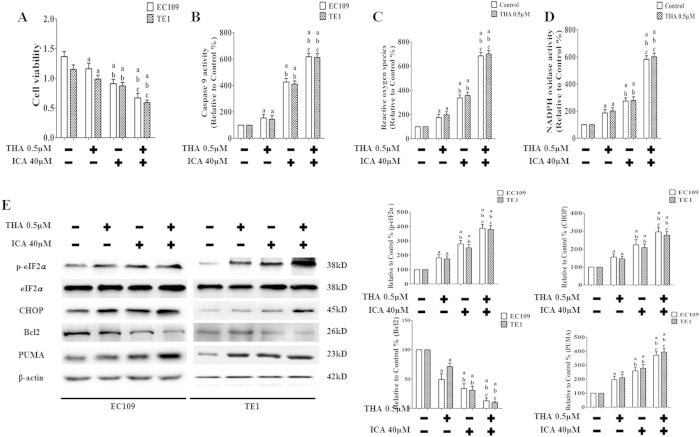
Effect of ICA combined with THA on cell viability, Caspase 9 activity, ROS induction, NADPH oxidase activity, and p-eIF2α levels in human ESCC cells. (**A**) Viability is expressed as OD values. (**B**) The intracellular Caspase 9 activity levels are shown. (**C**) ROS concentrations are shown. (**D**) NADPH oxidase activity is shown. The two indexes in the control group were defined as 100%. (**E**) Representative Western blot results of p-eIF2α, CHOP, Bcl2, and PUMA are shown. Membranes were re-probed for β-actin expression to show that similar amounts of protein were loaded in each lane. The results are expressed as the mean ± SD; n = 6. ^a^P < 0.05 *vs.* the control group; ^b^P < 0.05 *vs.* the THA 0.5 μM-treated group; ^c^P < 0.05 *vs.* the ICA 40 μM-treated group.

THA, an irreversible inhibitor of sarcoplasmic reticulum/ER calcium ATPase (SERCA), induces calcium signaling and the UPR, thereby significantly decreasing cancer cell viability via apoptosis^33^,^34^. We combined ICA (40 μM) with THA and determined the cytotoxicity to EC109 and TE1 cells. The concentration and duration of THA treatment (0.5 μM for 24 h) that effectively enhanced ERS activity without affecting cell viability were selected based on our preliminary experiments. As shown in Fig. 8A, the OD value in the ICA treatment group was 0.91 ± 0.07 in EC109 cells and 0.87 ± 0.06 in TE1 cells, and THA treatment alone slightly increased the OD value to 1.16 ± 0.09 in EC109 cells and 0.98 ± 0.07 in TE1 cells. However, when ICA was combined with THA, the OD value was significantly decreased to 0.67 ± 0.08 in EC109 cells and 0.59 ± 0.04 in TE1 cells. The combination index (CI) was calculated according to a previous study^35^; CI < 1 indicates synergistic activity, CI = 1 indicates additive activity, and CI > 1 indicates antagonistic activity. The CIs in the two ESCC cell lines used in this study were approximately less than 1 (0.86 in EC109 and 0.79 in TE1), indicating that the combination of ICA and THA exerted a synergistic effect. The combination of ICA and THA significantly increased Caspase 9 activity (Fig. 8B), ROS generation (Fig. 8C), and NADPH oxidase activity (Fig. 8D) (P < 0.05 compared with ICA or THA alone). As shown in Fig. 8E, THA significantly increased PUMA expression in ESCC cells (P < 0.05 compared with the control), and treatment with both ICA and THA in EC109 and TE1 cells increased PUMA expression compared with either treatment alone (P < 0.05). Moreover, treatments of ICA and THA upregulated p-eIF2α and CHOP and downregulated Bcl2 (P < 0.05 compared with ICA or THA treatment alone). Additionally, both N-linked glycosylation inhibitor tunicamycin (TM)^36^,^37^ and strong reducing agent dithiothreitol (DTT)^38^, another two ERS inducers, further upregulated p-PERK, CHOP, and PUMA and downregulated Bcl2 (P < 0.05 compared with ICA, TM, or DTT treatment alone) (Supplementary Fig. 7).

### Effects of ICA on tumor xenograft growth and ERS signaling *in vivo*

**Figure 9:**
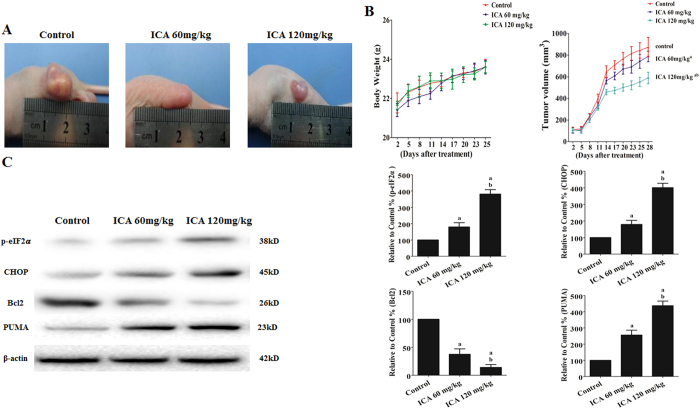
Effect of ICA on EC109 tumor xenografts *in vivo*. (**A**) Photographs showing tumor xenograft morphologies in each group. (**B**) Change in body weight of the mice. A tumor growth curve was drawn from the tumor volumes and treatment duration. (**C**) Representative Western blot results of p-eIF2α, CHOP, Bcl2, and PUMA are shown. Membranes were re-probed for β-actin expression to show that similar amounts of protein were loaded in each lane. The results are expressed as the mean ± SD; n = 6. ^**a**^P < 0.05 *vs.* the control group; ^**b**^P < 0.05 *vs.* the 60 mg/kg ICA-treated group.

*In vivo* tumor xenograft experiments, our results *in vitro* were further verified. To determine whether ICA inhibits tumor growth in animals, we established an EC109 cell-based tumor-bearing model using athymic nude mice. We found that the mice in all of the treatment groups developed subcutaneous tumors. In the pre-experiment, the drug concentration in the serum of mice with intraperitoneally administered 90 mg/kg of icariin daily for five days reached a peak at about 39.7 μM by a high performance liquid chromatography assay^39^, which just fell within the range of the drug concentrations (20–80 μM) *in vitro*. So 60 and 120 mg/kg of ICA were selected for *in vivo* experiments. When transplant tumors reached a mean group size of approximately 100 mm^3^, mice were treated every day for 20 days with various dose of icariin (i.p., 60 and 120 mg/kg). As shown in Fig. 9A, ICA treatment (60 or 120 mg/kg) significantly inhibited tumor growth (P < 0.05 compared with the control treatment). The body weight and tumor size of the mice are shown in Fig. 9B. Western blot analysis indicated that ICA treatment induced a dose-dependent upregulation of GRP78 and CHOP. Furthermore, the anti-apoptotic protein PUMA was upregulated by ICA treatment, whereas Bcl2 was downregulated by ICA treatment (P < 0.05 compared with the control treatment, Fig. 9C).

## Materials and Methods

### Drugs and reagents

ICA, dimethyl sulfoxide (DMSO), 3-(4,5-dimethylthiazol-2-yl)-2,5-diphenyltetrazolium bromide (MTT), 4’,6-diamidino-2-phenylindole (DAPI), thapsigargin (THA, an ERS inducer), tunicamycin (TM), and 2’,7’-dichlorofluorescein diacetate (DCFH-DA) were purchased from Sigma-Aldrich (St. Louis, MO, USA). A Cell Counting Kit-8 (CCK-8) was purchased from Dojindo (Kumamoto, Japan). Dithiothreitol (DTT) was purchased from Fisher Scientific (Pittsburgh, PA, USA). eIF2α small interfering RNA (siRNA) and antibodies against ATF4, GRP78, CHOP, PERK, eIF2α, and β-actin were obtained from Santa Cruz Biotechnology (Santa Cruz, CA, USA). Antibodies against phosphorylated eIF2α (p-eIF2α), phosphorylated PERK (p-PERK), Bcl2, and PUMA were obtained from Cell Signaling Technology (Beverly, MA, USA). Terminal deoxynucleotidyl transferase dUTP nick-end labeling (TUNEL) kits were purchased from Roche Diagnostics (Mannheim, Germany). A Caspase 9 Cellular Activity Assay kit was purchased from R&D Systems (Minneapolis, MN, USA). A glutathione (GSH) assay kit was obtained from Shanghai Enzyme-linked Biotechnology Co., Ltd. (Shanghai, China). Goat anti-rabbit, goat anti-mouse, and rabbit anti-goat secondary antibodies were purchased from CMCTAG Company (Milwaukee, WI, USA).

### Cell culture

Two human ESCC cells lines (EC109 and TE1 cells) were provided by the Cancer Institute of the Chinese Academy of Medical Sciences (Beijing, China). Immortalized human primary esophageal epithelial HET-1A cell were purchased from American Type Culture Collection (Manassas, Virginia, USA). The ESCC cells were routinely cultured in Dulbecco’s modified Eagle’s medium (DMEM, Gibco, Grand Island, NY, USA); and HET-1A cell was grown in RPMI-1640 medium (BioWhittaker, Walkersville, MD, USA) at 37 °C in a humidified atmosphere containing 5% CO_2_. All the media were supplemented with 10% fetal bovine serum (FBS, Gibco), L-glutamine (2 mM), penicillin (100 units/ml), and streptomycin (100 units/ml) (Invitrogen, Breda, Netherlands) according to the provider’s instructions^40^.

### Drug treatments

ICA was dissolved in DMSO to create the stock solution, which was diluted in culture media immediately prior to an experiment. The concentration of DMSO was less than 0.1%. FBS-free DMEM or RPMI-1640 containing same volume of DMSO as above was used as the control. The ESCC cells were treated with ICA (20, 40, or 80 μM) in the first part of our study and then treated with ICA (40 μM) in the absence or presence of three ERS inducers [THA (0.5 μM), TM (3 μM), and DTT (1 μM)] and eIF2α siRNA (pretreated for 24 h) for different periods of time (as determined based on the preliminary experiments) in the second part of our study. After the different treatments, the cells were harvested for further analysis. The HET-1A cells was administrated with ICA (20, 40, or 80 μM), similarly.

### Analysis of cellular apoptosis

EC109 and TE1 cell apoptosis was analyzed using an *In Situ* Cell Death Detection Kit according to the manufacturer’s instructions, and the results were obtained using a fluorescence microscope. Briefly, after the different treatments, the two ESCC cell lines grown on cover slips were washed twice with PBS and fixed in 4% paraformaldehyde for 30 min; subsequently, endogenous peroxidase activity was inhibited by incubation in 3% hydrogen peroxide diluted in methanol for 10 min at room temperature. The cells were then permeabilized using 0.1% Triton X-100 for 5 min on ice. After washing with PBS, the cells were covered with 75 μL of the TUNEL reaction mixture, and 2 negative controls were generated using 75 μL of Label Solution per sample. All of the cell samples were incubated in this solution for 60 min at 37 °C in a humidified dark chamber. Finally, the nuclei were stained with DAPI-containing mounting medium prior to visualization using a BX51 light microscope (Olympus, Japan). The TUNEL-positive cells, which displayed green nuclear staining, and all of the cells displaying blue nuclear DAPI staining were counted in 5 randomly selected fields under high-power magnification. The apoptosis index was calculated as the ratio of TUNEL-positive apoptotic cells to the total number of cells counted × 100%.

### Analysis of cell viability

The CCK-8 assay was employed to quantitatively evaluate cell viability. Cultured EC109 and TE1 cells were carefully washed twice with PBS and then harvested using 0.25% trypsin. The cells were collected via centrifugation at 800 rpm for 3 min. Next, the cells were seeded on 96-well plates at a density of 6,000 cells per well in 100 μL of DMEM supplemented with 10% FBS; five parallel replicates were prepared and were exposed to various treatments. The control group was treated with DMEM containing 0.1% DMSO. Next, 10 μL of CCK-8 solution was added to each well, and the plates were incubated at 37 °C in a 5% CO_2_/95% air humidified incubator for 2 h. Optical density (OD) values were obtained at 450 nm using a microplate reader (SpectraMax 190, Molecular Device, USA), and cell viability was expressed as the OD value^41^. In addition, cell morphology was analyzed using an inverted/phase contrast microscope, and images were acquired using a 600D camera (Canon Company, Japan). All experiments were repeated three times.

### Wound healing assay and cell adhesion analysis

As described in previous studies^42^,^43^, migration and adhesion should be studied under conditions that do not affect cell activity to determine their effect on the invasiveness and metastasis of cancer. In our preliminary experiments, we found that ICA treatment at concentrations lower than 20 μM for 24 h did not affect the proliferation of the two ESCC cells lines (Supplementary Fig. 1A–C). Therefore, we performed adhesion and migration assays after 24 h of treatment with ICA (5, 10, or 15 μM) as previously described^44^. After treatment, the cells were collected using 0.25% trypsin and then resuspended in basal medium containing 10% fetal bovine serum. Subsequently, 100 μL of medium containing 1 × 10^4^ cells was added to each well of a 96-well plate, and the cells were placed in the incubator for 0.5 h at 37 °C. Thereafter, the medium in each well was discarded. Next, the wells were washed three times with PBS, and the adherent cells were stained with MTT. The stained cells were observed using an inverted phase-contrast microscope. Images were acquired using a 600D camera (Canon, Japan), and five fields were randomly selected for quantification. Finally, 100 μL of DMSO was added to each well, and the plates were incubated for 30 min at 37 °C with shaking. The OD value of each well at 490 nm was measured using a SpectraMax 190 spectrophotometer (Molecular Devices, Sunnyvale, CA, USA), and the OD value of the control group was normalized to 100%.

The resuspended cells were seeded on a 6-well plate in basal medium containing 10% FBS and were cultured at 37 °C with a humidified 5% CO_2_ incubator to enable the formation of cell monolayers. After the cells grew to confluence, an artificial wound was applied by scratching the dish with a P200 pipette tip. The cells were then washed twice with PBS to remove cellular debris, and different concentrations of ICA (5, 10, or 15 μM) were added for 24 h. The wound edge movement was monitored under a microscope, and the results were expressed as the distance between the sides of the scratch. The distance in the control group was set to 100%.

### Analysis of Caspase 9 activity

Caspase 9 activity was measured using a colorimetric assay kit according to the manufacturer’s recommendations. The cells were washed in ice-cold PBS, and the proteins were extracted and stored at −80 °C. The protein concentrations in the supernatants were determined using the Bradford assay (Bio-Rad, Hercules, CA, USA). After centrifugation at 10,000 × g at 4 °C for 20 min, the protein samples (20 μL) were subjected to an enzymatic reaction with 5 μL of Caspase 9 colorimetric synthetic substrate (LEHD-pNA). Next, buffer was added to yield a 100-μL total reaction volume, and this mixture was incubated at 37 °C for 2 h. The released pNA concentrations were calculated based on the absorbance values at 405 nm and the calibration curve of the standard pNA solutions. The degree of Caspase 9 activation was determined based on comparison with the level of Caspase 9 activation in the control group, which was normalized to 100%. The activity was expressed as the fold change relative to the control.

### Analyses of intracellular ROS generation, NADPH oxidase activity assay, and GSH levels

After treatment, the cells were trypsinized and subsequently incubated in DCFH-DA (5 μM) in PBS at 37 °C for 2 h. After washing the cells with PBS, the DCFH fluorescence of the cells in each well was measured using an FLX 800 fluorescence microplate reader at an excitation of 488 nm and an emission of 522 nm (Biotech Instruments, Inc., USA). A cell-free measurement was used as the background, and the fluorescence intensity in the control group was defined as 100%.

The generation of intracellular reduced GSH, which is an index of the cellular reducing power, was simultaneously measured using appropriate kits according to the provided instructions. Briefly, mitochondria were lysed in the presence of iodoacetic acid (10 mM) and were derivatized with fluorescent dansyl chloride. The derivatized samples were separated and analyzed via hydrophilic interaction liquid chromatography using a Restek Ultra Amino 3-μm 100 × 3.2 mm HPLC column at a flow rate of 0.6 mL/min. Buffer A (80% methanol/20% water) was mixed in a linear gradient of up to 30% of buffer B (acetate-buffered (pH 4.6) methanol solution) to elute GSH using a Varian ProStar 230 HPLC system. Fluorescent products were measured using an FLX 800 fluorescence microplate reader at an excitation of 335 nm and an emission of 541 nm. The GSH level in the control group was normalized to 100%.

The NADPH oxidase activity assay was performed by modifying the method described by previous study^45^. Briefly, the cells after different treatments were harvested in Krebs-HEPES buffer (pH 7.4) containing protease and phosphatase inhibitor cocktail, homogenized with Dounce homogenizer, and centrifuged at 10,000 g for 15 min. After determining protein concentration using a BCA protein assay kit (Thermo Scientific, Life Technologies, Carlsbad, CA, USA), equal amount of proteins was transferred to the 96 well plate with 10 μM lucigenin prepared in the same buffer and incubated at 37 °C for 10 min. The chemiluminescence was measured over the subsequent 1 min in response to the addition of 100 μM NADPH using the luminescence channel of a Fluostar Optima microplate reader (BMG Labtech, Offenburg, Germany). Data are expressed as relative levels in the control group and the NADPH oxidase activity in the control group was normalized to 100%.

### siRNA transfection

For the siRNA transfections, ESCC cells were seeded on 6-well plates at 2 × 10^5^ cells per well in 2 mL of antibiotic-free normal growth medium supplemented with FBS. When the cells reached 70–80% confluence, they were transiently transfected with 200 μL of negative control or eIF2α siRNA and 800 μL of siRNA transfection medium for 6 h. Next, 1 mL of normal DMEM containing 20% FBS was added to each well without removing the transfection mixture. After incubating the cells for an additional 24 h, the medium was aspirated, and fresh DMEM containing 10% FBS was added for 24 h. Finally, the cells were prepared for use in further experiments.

### Anticancer activity in a xenograft model

Male athymic nude mice were purchased from the Laboratory Animal Centre of the Fourth Military Medical University. The mice were fed and maintained under specific pathogen-free conditions in facilities approved by the American Association for Accreditation of Laboratory Animal Care and in accordance with the current regulations and standards of the United States Department of Agriculture and the United States Department of Health and Human Services. All of the operations were performed according to the Guide for the Care and Use of Laboratory Animals published by the United States National Institutes of Health (Publication No. 85-23, revised 1996) and were approved by the Ethics Committee of The Fourth Military Medical University. All of the mice were anesthetized using sodium pentobarbital, and all efforts were made to minimize animal suffering. EC109 cell-based tumor xenografts were established by subcutaneously injecting 1 × 10^7^ cells into the left hind limb of 4–6-week-old athymic nude mice. The tumor volume (V) was calculated using the following formula: V = L × W^2^/2 (L = largest diameter; W = smallest diameter). Based on the data from a preliminary study, we initiated treatment when V reached approximately 100 mm^3^, at which point the mice were randomly allocated to 3 groups (n = 6 per group): the control group (0.05% DMSO) and two ICA treatment groups. ICA was diluted in saline and DMSO and was administered intraperitoneally every day for 20 days. The body weight and tumor size were measured every 3 days using calipers (on days 2, 5, 8, 11, 14, 17, 20, 23, 25, and 28); on day 28, the tumors were excised from the euthanized mice for Western blot analysis.

### Western blot

The cells or tumor samples were scraped or ground, respectively, and then lysed in sample buffer [150 mM Tris (pH 6.8), 8 M urea, 50 mM DTT, 2% sodium dodecyl sulfate, 15% sucrose, 2 mM EDTA, 0.01% bromophenol blue, and 1% protease and phosphatase inhibitor cocktail], sonicated, boiled, separated on a 8–12% Bis/Tris gel using 5 × MES buffer (Invitrogen) and transferred to an Immobilon nitrocellulose membrane (Millipore, Billerica, MA, USA). The membranes were blocked with 5% BSA in TBST [150 mM NaCl, 50 mM Tris (pH 7.5), and 0.1% Tween-20] and then probed using antibodies against p-PERK, PERK, eIF2α, ATF4, GRP78, CHOP (1:500 for each), p-eIF2α, Bcl2, PUMA, and β-actin (1:1000 for each) for 18 h at 4 °C. Next, the membranes were placed in blocking buffer, washed with TBST, probed using secondary antibodies (1:5000) in blocking buffer at room temperature for 2 h and then washed. The fluorescent signal was detected using a BioRad imaging system (BioRad, Hercules, CA, USA), and the signal was quantified using Image Lab Software (BioRad, Hercules, CA, USA).

### Statistical analyses

All of the data are presented as the means ± standard deviation (m ± SD). Between-group comparisons were performed using ANOVA (SPSS 13.0). All of the groups were analyzed simultaneously using the LSD test. A P value of less than 0.05 was considered to be significant.

## Discussion

Esophageal cancer is the sixth most common cause of cancer-related deaths worldwide. The clinical outcomes of esophageal cancer are poor, and diagnosis at an earlier disease stage is associated with improved outcome. Surgical resection using various techniques is the first-line treatment for early-stage esophageal cancer, resulting in an overall 5-year survival rate ranging from 10% to 36%^46^. Therefore, it is important to investigate the molecular mechanisms underlying esophageal cancer development and to identify effective and non-cytotoxic chemical agents for esophageal cancer chemoprevention and treatment. For example, APR-246 upregulated p53 target genes, inhibited clonogenic survival and induced cell cycle arrest as well as apoptosis in oesophageal adenocarcinomas cells harbouring p53 mutations^47^. In other respect, many investigators are studying natural active compounds for cancer chemoprevention and treatment^6^.

ICA is one of the major bioactive constituents of Herba Epimedii which is mainly metabolized via desugarization, dehydrogenation, hydrogenation, dehydroxylation, hydroxylation, demethylation and glucuronidation pathways in rodent^48^. As a natural prenylated flavonol glycoside, ICA, at the level of μM concentration, has been shown to suppress the proliferation of various types of cancer cells^12^,^13^,^14^,^49^. Various molecules and signaling pathways are involved in the antitumor effects of ICA. For example, the ICA-induced apoptosis of human hepatoma SMMC-7721 cells is mediated by the activation of the mitochondrial apoptotic pathway, which requires ROS generation and JNK activation^39^. Icariin sensitizes cells to TRAIL-induced apoptosis by suppressing NF-κB-dependent c-FLIP expression^50^. Furthermore, icariin treatment decreases PIWIL4 protein expression, which increases apoptosis by regulating the expression of Bcl2/Bax and cytochrome c and the activation of Caspase 9 and 3 in MLTC-1 cells^30^. Although there have been reports concerning the anticancer effect of icariin in some tumors, the effect of icariin on human esophageal cancer and the mechanisms responsible for these effects are not fully understood. In the present study, ICA treatment resulted in the dose- and time-dependent induction of apoptosis and reduction in cell viability in EC109 and TE1 cells. In EC109 and TE1 cells, ICA also significantly decreased adhesion and migration, both of which are major events related to tumor metastatic potential. Additionally, ICA treatment markedly inhibited tumor growth in an EC109 cell-based xenograft nude mouse model.

Proteins enter the secretory pathway at the ER for trafficking to the cell surface and to intracellular organelles. Protein folding in the ER is highly regulated, and only properly folded proteins perform their physiological functions^17^. Because of their high growth and proliferation rates, cancer cells require an increased rate of protein folding and assembly in the ER. In addition, some cancer cells express mutant proteins that cannot be correctly folded, which activates the UPR^51^. ERS generally plays a protective role in tumor development by activating adaptive stress response elements and by attenuating apoptotic pathways. However, some studies have shown that the therapeutic induction of ERS-induced apoptosis might be beneficial for killing cancer cells. For example, a selective proteasome inhibitor, bortezomib, has been reported to effectively kill multiple myeloma cells and to exhibit antitumor activity in pancreatic cancer^52^,^53^. In our study, we explored the role of ERS in the anticancer activity of ICA in human ESCCs. Our results indicated that ICA treatment upregulated ATF4, GRP78, and CHOP expression and p-PERK and p-eIF2α levels in EC109 and TE1 cells. Furthermore, ICA treatment significantly induced p-eIF2α and CHOP expression in EC109 cell-based xenografts. *In vitro* siRNA-mediated silencing of the ERS response protein eIF2α significantly reduced ICA-induced cell death. In contrast, the anticancer activity of ICA was synergistically enhanced by THA, TM, or DTT, which effectively activated ERS. These data suggested that the anticancer activity of ICA in human ESCCs was mediated, at least in part, by the activation of ERS signaling.

The mitochondria-initiated apoptotic pathway is the predominant apoptotic pathway in mammalian cells and is tightly regulated by Bcl2 family proteins, such as Bcl2 and PUMA (the key regulators of apoptosis). Generally, Bcl2 family proteins establish connections with the UPR at the ER and maintain the homeostasis of cellular apoptotic programs^54^. Bcl2 protects against apoptosis by reducing the release of Ca^2+^ from the ER and the translocation of the pro-apoptotic protein Bax to the mitochondria, thereby suppressing apoptosis^55^. However, PUMA has recently been identified to be markedly induced by ERS in human neuroblastoma cells, and PUMA may contribute to ERS-induced apoptosis in human colon cancer cells^56^. PUMA, as a target and mediator of p53-mediated apoptosis, is a pro-apoptotic protein that is physiologically expressed at a low level; however, PUMA expression is markedly induced after cellular exposure to DNA-damaging agents, thereby promoting cytochrome c release^57^. Cytochrome c binds to Apaf-1 and Caspase 9, resulting in the activation of Caspase 9, the subsequent activation of Caspase 3 and Caspase 7 and, ultimately, cell death^58^. Our results indicated that ICA treatment downregulated Bcl2 expression and upregulated PUMA expression and Caspase 9 activity in EC109 and TE1 cells. In addition, our experiments confirmed that the inhibition of ERS using eIF2α siRNA reversed the effects of ICA on the expression of Bcl2 and PUMA, whereas treatment with the ERS inducers (THA, TM, or DTT) enhanced the effects of ICA on Bcl2 and PUMA expression. These results revealed that ICA induces apoptosis via the Bcl2 family pathway, which is consistent with the results of previous studies that suggested that the activation of ERS signaling is associated with the induction of the mitochondrial apoptotic pathway in human cancer cells^57^,^59^.

As multifunctional signaling molecules that participate in several cellular processes, ROS play important roles in the determination of cell fate, primarily in the mitochondria^60^. Recently, ROS have been identified as potential targets of novel anticancer drugs; ROS accumulation may represent the cause or a result of increased ERS^61^. The most important multi-enzyme complex involved in the generation of ROS mainly during signal transduction pathways is a membrane-bound NADPH oxidase that elevates ROS within a few minutes after cell stimulation^62^. In our study, the apoptotic effects of ICA were associated with a rapid increase in the intracellular ROS levels and NADPH oxidase activity in EC109 and TE1 cells. Furthermore, the inhibition of ERS using eIF2α siRNA attenuated ICA-induced ROS production and NADPH oxidase activity; in contrast, the activation of ERS using THA enhanced ICA-induced ROS generation and NADPH oxidase activity. Tumor cells are significantly more sensitive than nonmalignant cells to changes in the levels of GSH, a major antioxidant of intracellular ROS; tumor cells exhibit an increased basal level of ROS-mediated signaling, which contributes to their increased rates of growth, metabolism and proliferation. Therefore, tumor cells may be more vulnerable to oxidative stress^63^, and the extent of exposure to ROS and of perturbation of the GSH redox balance play critical roles in determining whether cells undergo pro-survival or pro-death processes. Similarly, our study revealed that ICA-induced ROS generation was significantly associated with the consumption of intracellular GSH. Based on the above results, we suggest that ICA may exhaust the total cellular antioxidant capacity and increase the ROS levels beyond a certain threshold, which may contribute to the induction of apoptosis in human ESCCs.

**Figure 10:**
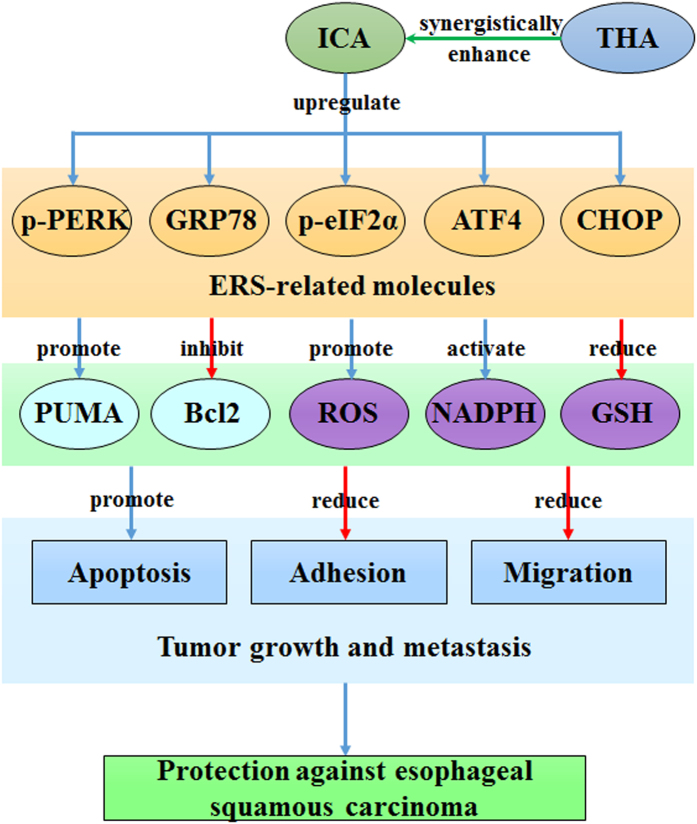
The ERS-dependent mechanism of action of ICA in human ESCC. ICA upregulates a series of ER-related molecules, including p-PERK, GRP78, p-eIF2α, ATF4, and CHOP. The activation of ERS signaling upregulates the expression of the apoptotic protein PUMA and downregulates the expression of the anti-apoptotic protein Bcl2. NADPH oxidase are activated. ROS are generated, and the intracellular GSH levels are reduced. Together, these events may induce apoptosis and may reduce the adhesive and migratory capacities of ESCC cells, thereby protecting against human esophageal cancer. In addition, THA synergistically enhances the anticancer activity of ICA. ICA, icariin; ERS, endoplasmic reticulum stress; ROS, reactive oxygen species; NADPH, nicotinamide adenine dinucleotide phosphate; GSH, glutathione; THA, thapsigargin; PERK, PKR-like endoplasmic reticulum kinase; GRP78, glucose-regulated protein 78; eIF2α, eukaryotic translational initiation factor 2α; ATF4, activating transcription factor 4; CHOP, C/EBP homologous protein; PUMA, p53 upregulated modulator of apoptosis; Bcl2, B-cell lymphoma-2.

As shown in Fig. 10, these experiments provided mechanistic *in vivo* and *in vitro* evidence that ICA is a potent inhibitor of human ESCC growth via the activation of ERS signaling. The downregulation of ERS signaling desensitized human ESCCs to ICA treatment, and the upregulation of ERS signaling sensitized human ESCCs to ICA treatment. Additionally, this potentiation of chemosensitivity by ERS may be related to the upregulation of pro-apoptotic pathways and activation of ROS. Most importantly, the stimulation of ERS signaling may serve as a novel strategy for preventing the induction of cancer cell survival mechanisms in human ESCC. Furthermore, ICA displays potential as a therapeutic agent against advanced ESCC in the clinical setting.

## Additional Information

**How to cite this article**: Fan, C. *et al.* Icariin displays anticancer activity against human esophageal cancer cells via regulating endoplasmic reticulum stress-mediated apoptotic signaling. *Sci. Rep.* **6**, 21145; doi: 10.1038/srep21145 (2016).

## Supplementary Material

Supplementary Information
