# Supplementary material for: Icariin displays anticancer activity against human esophageal cancer cells via regulating endoplasmic reticulum stress-mediated apoptotic signaling
Source: Sci Rep. 2016 Feb 19;6:21145. doi: 10.1038/srep21145 (PMC4759694; doi:10.1038/srep21145)
Supplement: Supplementary Information [file srep21145-s1.docx]

**Supplementary Information**

**Title of manuscript:**

Icariin displays anticancer activity against human esophageal cancer cells via regulating endoplasmic reticulum stress-mediated apoptotic signaling

**Authors:**

Chongxi Fan, Yang Yang, Yong Liu, Shuai Jiang, Shouyin Di, Wei Hu, Zhiqiang Ma, Tian Li, Yifang Zhu, Zhenlong Xin, Guiling Wu, Jing Han, Xiaofei Li, and Xiaolong Yan

**Supplementary information includes:**

Supplementary Figs. S1-S7

**Supplementary Fig. 1 Effect of ICA treatment on the viability and morphology of human ESCC cells. A.** EC109 cells were treated with increasing concentrations of ICA (5, 15, or 15 µM) and assessed at different time points (12, 24, and 36 h). The cell viability is expressed as OD values. **B.** TE1 cells were treated with increasing concentrations of ICA (5, 15, or 15 µM) and assessed at different time points (12, 24, and 36 h). The cell viability is expressed as OD values. **C.** The morphology of both of the ESCC lines was observed under an inverted phase-contrast microscope after the cells were treated for 24 h, and images were obtained. All of the results are expressed as the mean ± SD; n = 6.

**
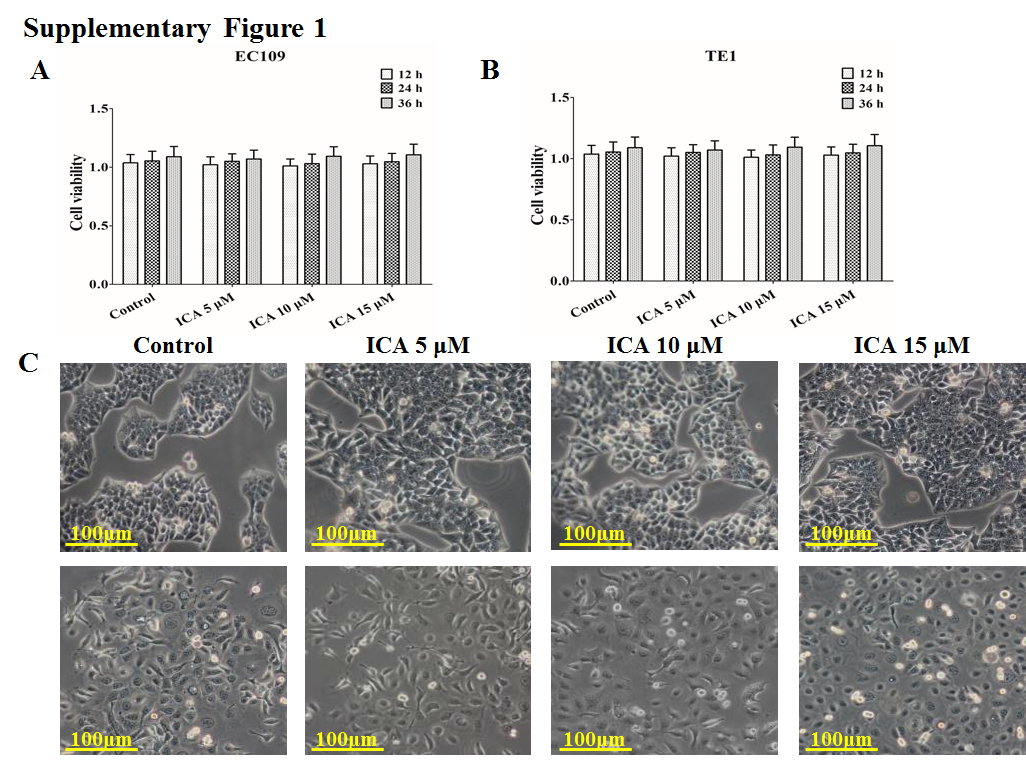
**

**Supplementary Fig. 2 Effect of ICA treatment on the viability and morphology of human** primary esophageal epithelial cell**.** HET-1A cells were treated with increasing concentrations of ICA (20, 40, or 80 µM) and assessed at different time points (12, 24, and 36 h). The cell viability is expressed as OD values. All of the results are expressed as the mean ± SD; n = 6.

**
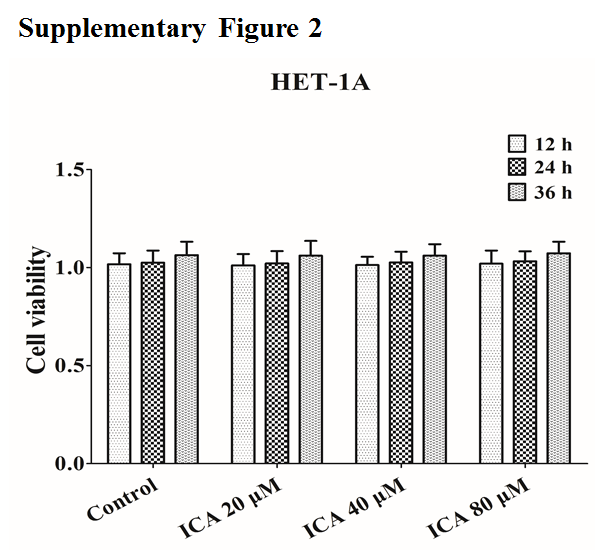
**

**Supplementary Fig. 3:** The full length blots in Fig. 6


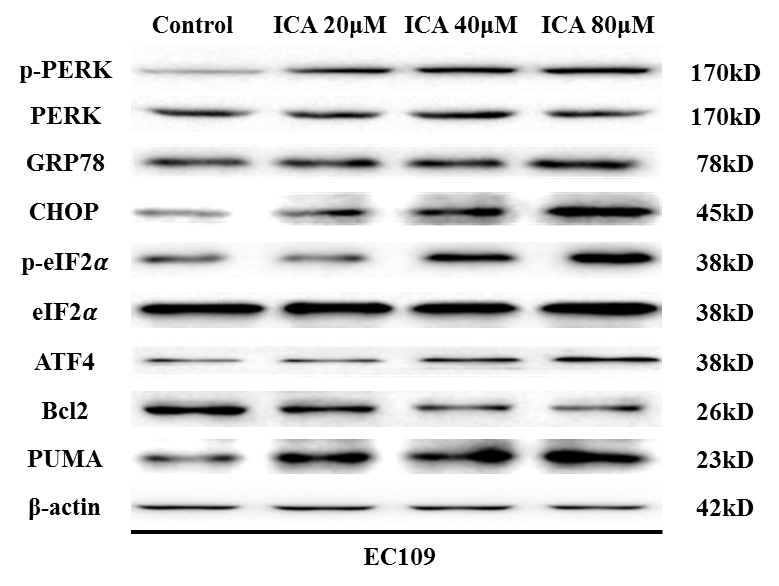


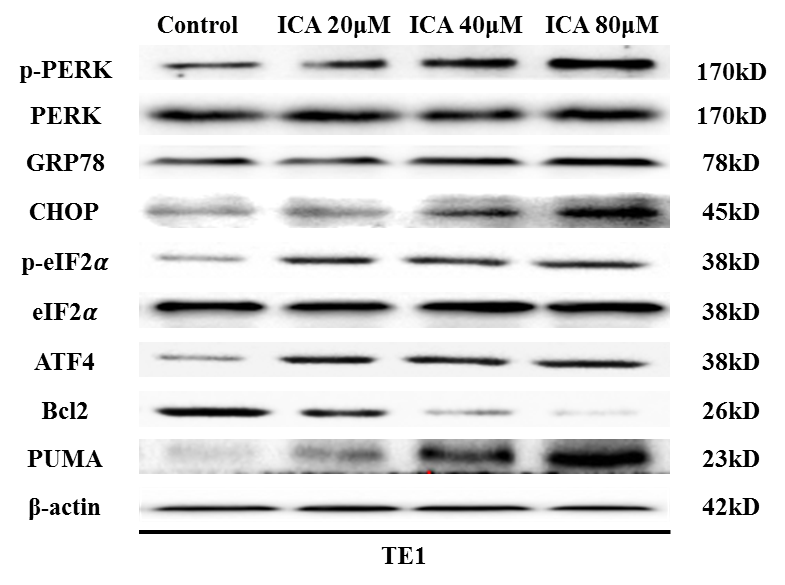


**Supplementary Fig. 4:** The full length blots in Fig. 7E


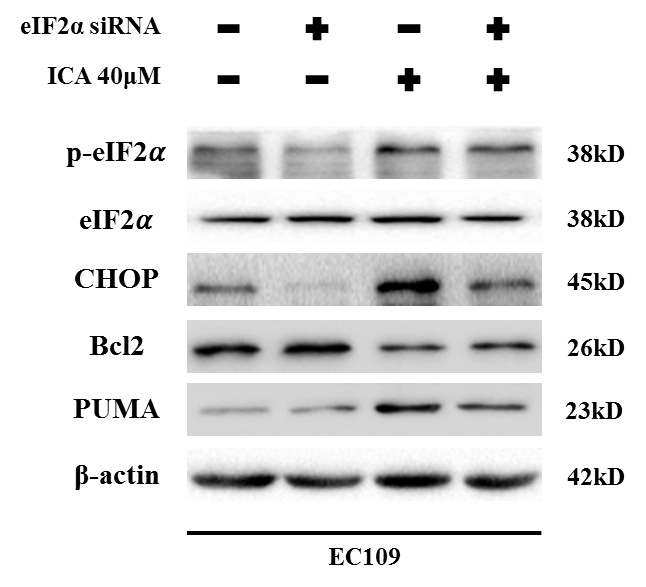


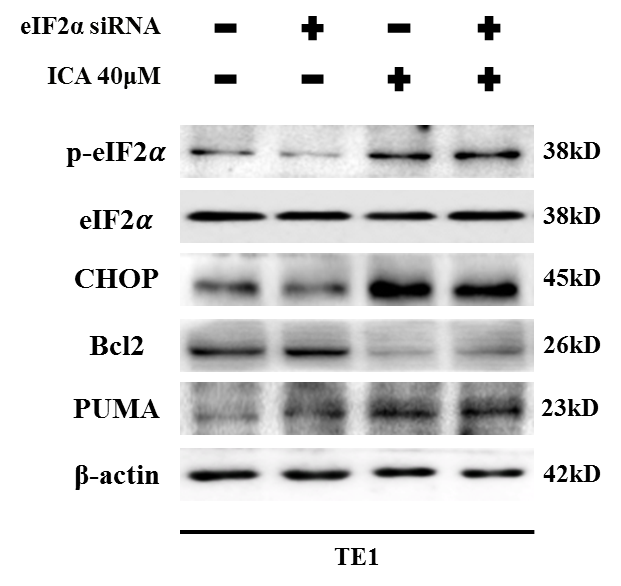


**Supplementary Fig. 5:** The full length blots in Fig. 8E


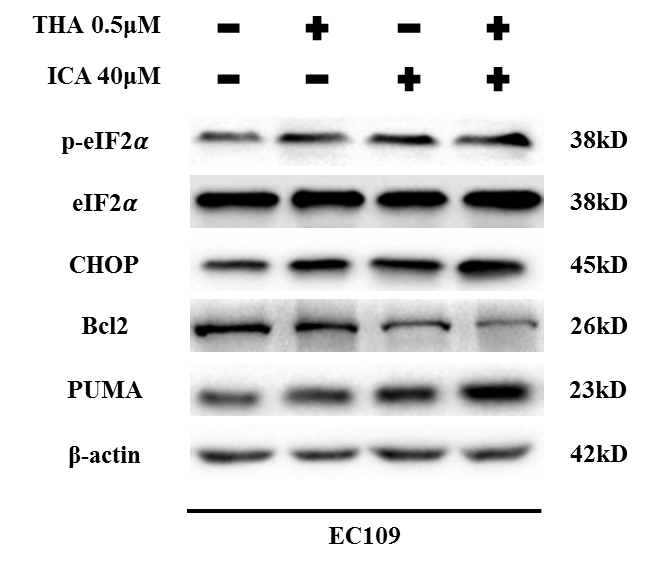


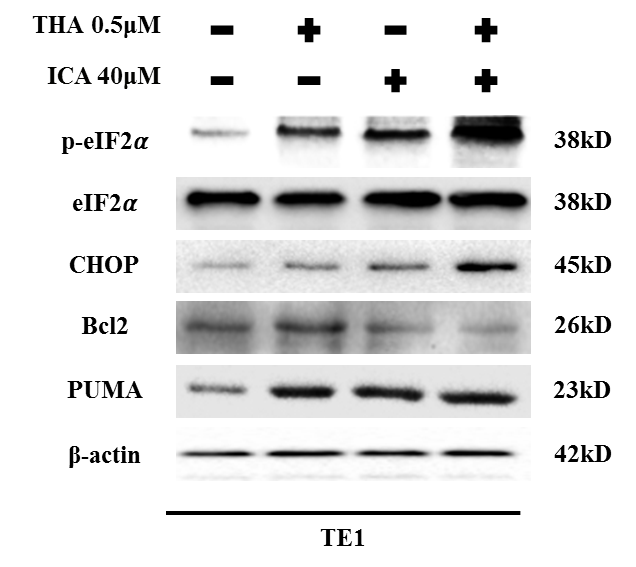


**Supplementary Fig. 6:** The full length blots in Fig. 9C


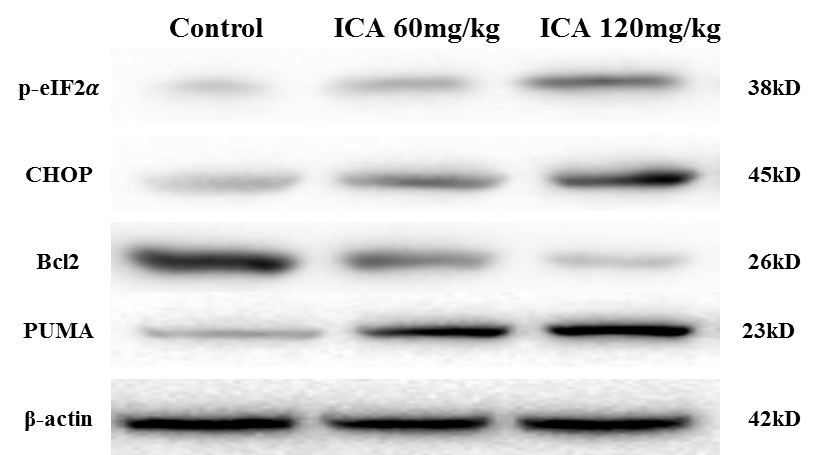


**Supplementary Fig. 7: Effect of ICA combined with TM or DTT on p-PERK, CHOP, Bcl2, and PUMA levels in human ESCC cells. A.** Representative Western blot results of p-PERK, CHOP, Bcl2, and PUMA after treated with ICA and TM are shown. **B.** Representative Western blot results of p-PERK, CHOP, Bcl2, and PUMA after treated with ICA and DTT are shown. Membranes were re-probed for β-actin expression to show that similar amounts of protein were loaded in each lane. The results are expressed as the mean ± SD; n = 6. **^a^**P < 0.05 *vs.* the control group; **^b^**P < 0.05 *vs.* the TM (3 μM) or DTT (1 μM)-treated group; **^c^**P < 0.05 *vs.* the ICA 40 μM-treated group.

**42kD**

**170kD**

**23kD**

**26kD**

**45kD**

**170kD**


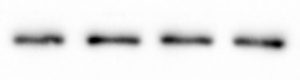

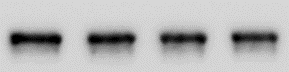

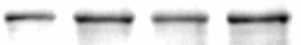

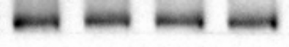

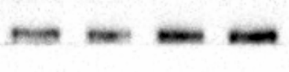


**β-actin**

**p-PERK**

**PUMA**

**Bcl2**

**CHOP**

**TM 3μM**

**ICA 40μM**

**PERK**

**EC109**


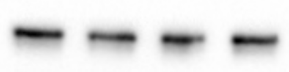

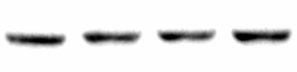

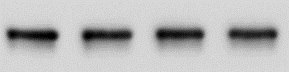

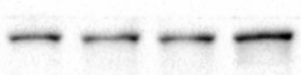

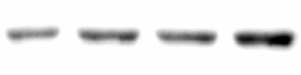

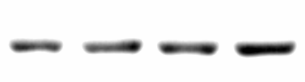


**β-actin**

**42kD**

**p-PERK**

**170kD**

**23kD**

**PUMA**

**Bcl2**

**26kD**

**45kD**

**CHOP**

**TM 3μM**

**ICA 40μM**

**PERK**

**170kD**

**TE1**


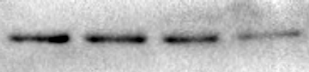

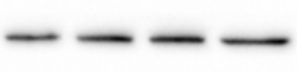

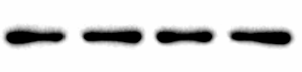

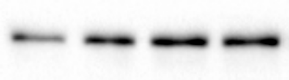

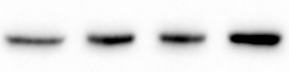

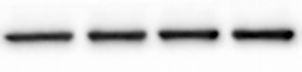


**β-actin**

**42kD**

**p-PERK**

**170kD**

**23kD**

**PUMA**

**Bcl2**

**26kD**

**45kD**

**CHOP**

**DTT 1μM**

**ICA 40μM**

**PERK**

**170kD**

**EC109**


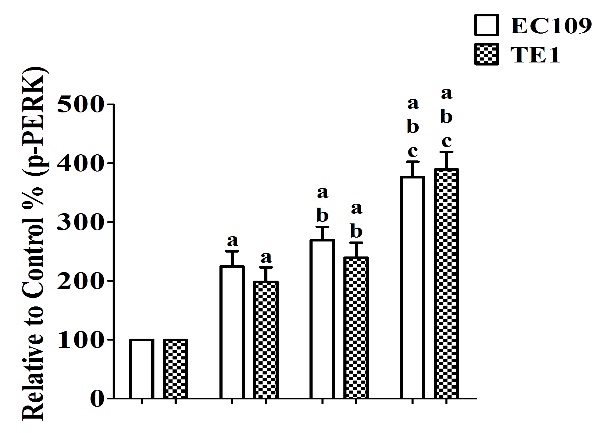


**TM 3μM**

**ICA 40μM**


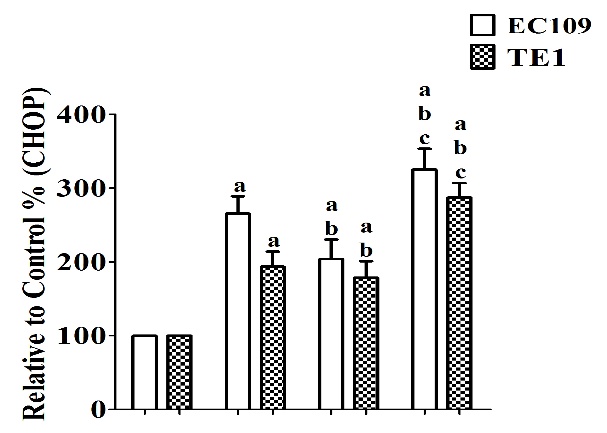


**TM 3μM**

**ICA 40μM**


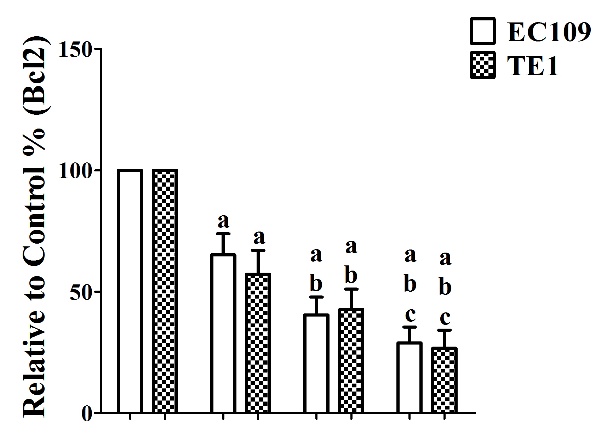


**TM 3μM**

**ICA 40μM**


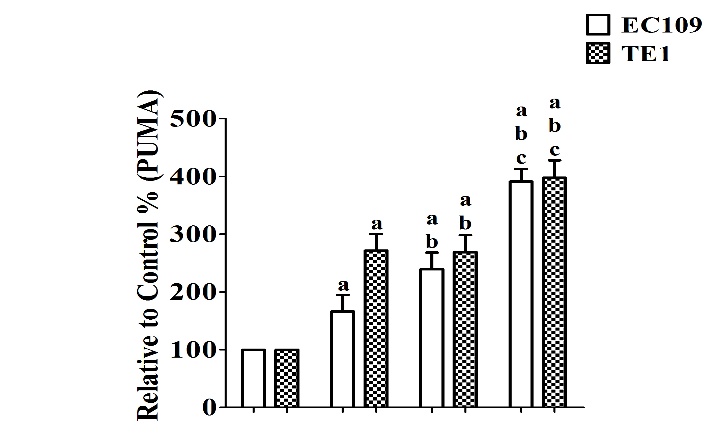


**TM 3μM**

**ICA 40μM**


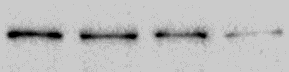

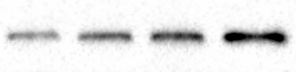

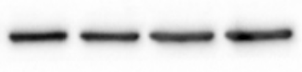

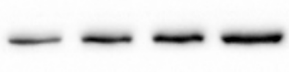

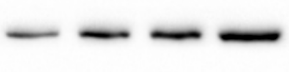

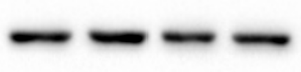


**β-actin**

**42kD**

**p-PERK**

**38kD**

**23kD**

**PUMA**

**Bcl2**

**26kD**

**45kD**

**CHOP**

**DTT 1μM**

**ICA 40μM**

**PERK**

**38kD**

**TE1**


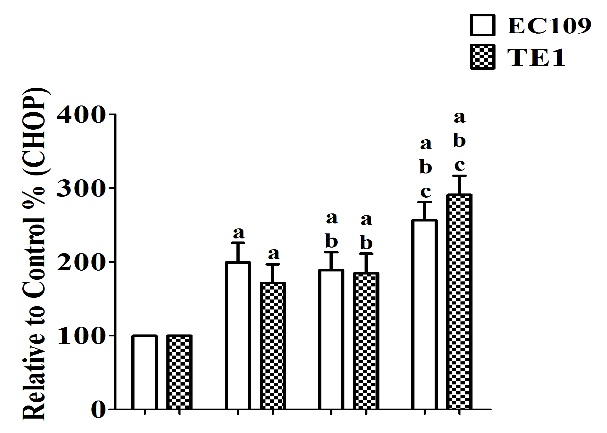

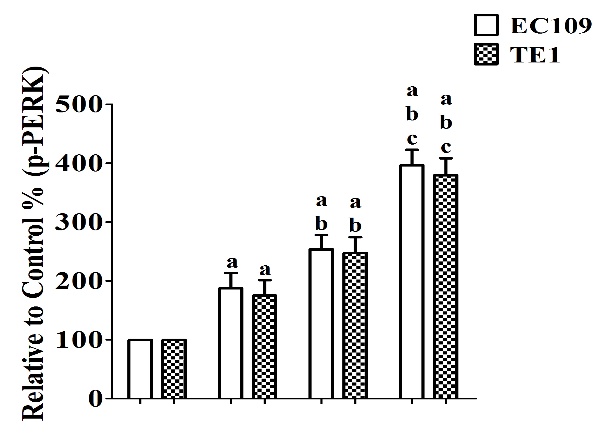

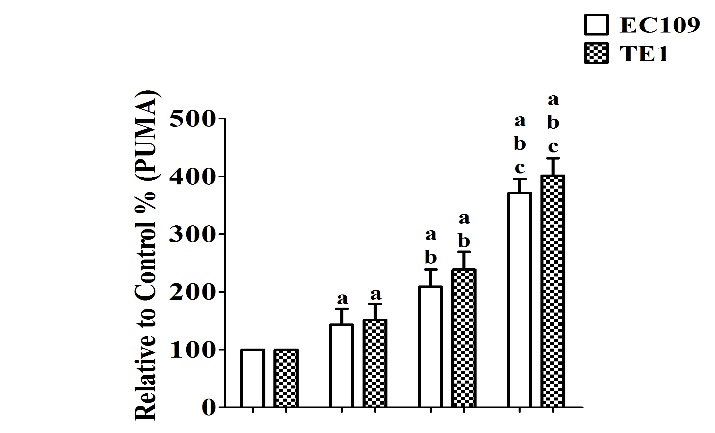

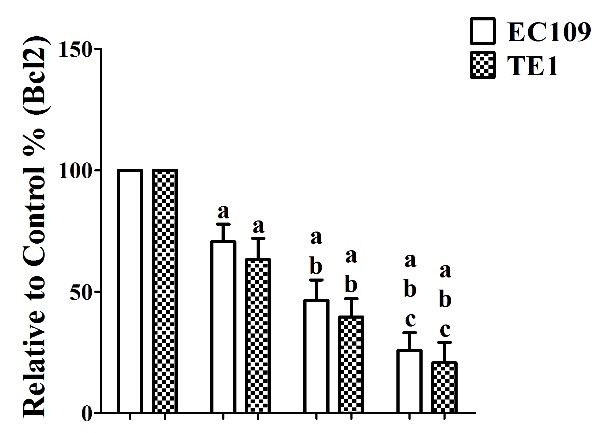


**DTT 1μM**

**ICA 40μM**

**DTT 1μM**

**ICA 40μM**

**DTT 1μM**

**ICA 40μM**

**DTT 1μM**

**ICA 40μM**
